# Lithium and brine geochemistry in the Qianjiang Formation of the Jianghan Basin, central China

**DOI:** 10.1038/s41598-023-31421-1

**Published:** 2023-03-17

**Authors:** Xiaocan Yu, Chunlian Wang, Hua Huang, Jiuyi Wang, Kai Yan

**Affiliations:** 1 Institute of Geology, Chinese Academy of Geological Sciences, Beijing, 100037 China; 2 MNR Key Laboratory of Metallogeny and Mineral Assessment, Institute of Mineral Resources, Chinese Academy of Geological Sciences, Beijing, 100037 China; 3 SINOPEC Jianghan Oilfield Company, Qianjiang, 433124 China

**Keywords:** Economic geology, Geochemistry, Hydrogeology

## Abstract

The Li-enriched oilfield brine is a very important lithium resource. It has gained much attention and become the target of active Li surveys with the growing global demand for Li. However, only little is known about their feature and nature. In the study, hydrochemical data from 155 oil wells tapping the Eocene to Lower Oligocene Qianjiang Formation of the Jianghan Basin, central China indicate that the brines are of the Na–Cl or Na–Ca–Cl type and are characterized by highly variable Li contents of 7.56 to 150 mg/L, with Mg/Li ratios less than 11.65. High Na/Cl and Cl/Br molar ratios indicate distinct contributions from halite dissolution. The Ca excess, Na deficit and Ca/Mg and Ca/Sr molar ratios in the brines imply multiple diagenetic processes, including halite dissolution, dolomitization, albitization and calcite or anhydrite cementation. The lithium contents of these brines have a weak relationship with the salinity and a negative correlation with Cl/Br ratios, possibly indicating that these Qianjiang oilfield brines have been diluted by secondary brines derived from halite dissolution. The spatial distribution patterns for Li and B concentrations of the brines are different from those for salinity and Br contents and show a geographic pattern, indicating that Li enrichment in the Qianjiang brines is likely connected with geothermal sources associated with volcanic activity.

## Introduction

The success of applications of Li batteries in electric and hybrid vehicles, in recent years, has spurred an increased interest in Li as a strategic resource, to the point that it has been served as one of the most critical metal minerals^1,2^. The principle lithium resources include Li-rich hard rocks (pegmatites and volcanogenic clays) and continental brines^3^, among which the latter accounts for about 65% of the global lithium occurrences^4^. These Li-rich brines with average Li grades > 600 mg/L are distributed in the salt pans of the South American Altiplano-Puna^5–7^, in the salt lakes of the Qinghai-Tibetan plateau^8–10^, and in the Great Basin of the western USA^11–13^.

As previously mentioned, the growing global demand for Li driven by its high electrochemical potential in emerging technologies and the rising price in the market greatly encourage the development of mining projects. As such, less-rich brine deposits such as oilfield brines and geothermal brines, with Li grades between 50 and 400 mg/L, gained much attention and became the target of active Li surveys throughout the world. Most petroliferous basins contain abundant Li-enriched oilfield brines^14^. The known oilfield brine deposits include those from the Jurassic Smackover Formation in the Gulf Coast Basin of America^15^, the Fox Creek area in the Alberta Basin of Canada^16^ and the Paleogene and Neogene formation in the western Qaidam Basin of China^17^.

The Jianghan Basin is an important Late Cretaceous to Oligocene petroliferous basin in central China. Large quantities of Li-rich oilfield brines are stored in the Upper Eocene to Lower Oligocene Qianjiang Formation of the basin. The brine deposits at mean Li grades of 60.6 mg/L hold LiCl resources of ~ 4 Mt^18,19^. Li enrichment in brines and evolution of brine hydrochemistry, however, remain unclear, except that potassium in these oilfield brines and reservoir characteristics have been reported by Yu et al.^20^.

This paper presents the first broad hydrochemical data of oilfield brines from the Qianjiang Formation of the Jianghan Basin, China and aims to investigate the water chemistry characteristics and temporal and spatial distribution of Li in brines and determine its potential enrichment mechanism. In addition, this study also offers an overview of the potential of unconventional Li survey and contributes to knowledge of the origin of Li-enriched oilfield brines in the continental petroliferous basins.

## Geological setting

**Figure 1:**
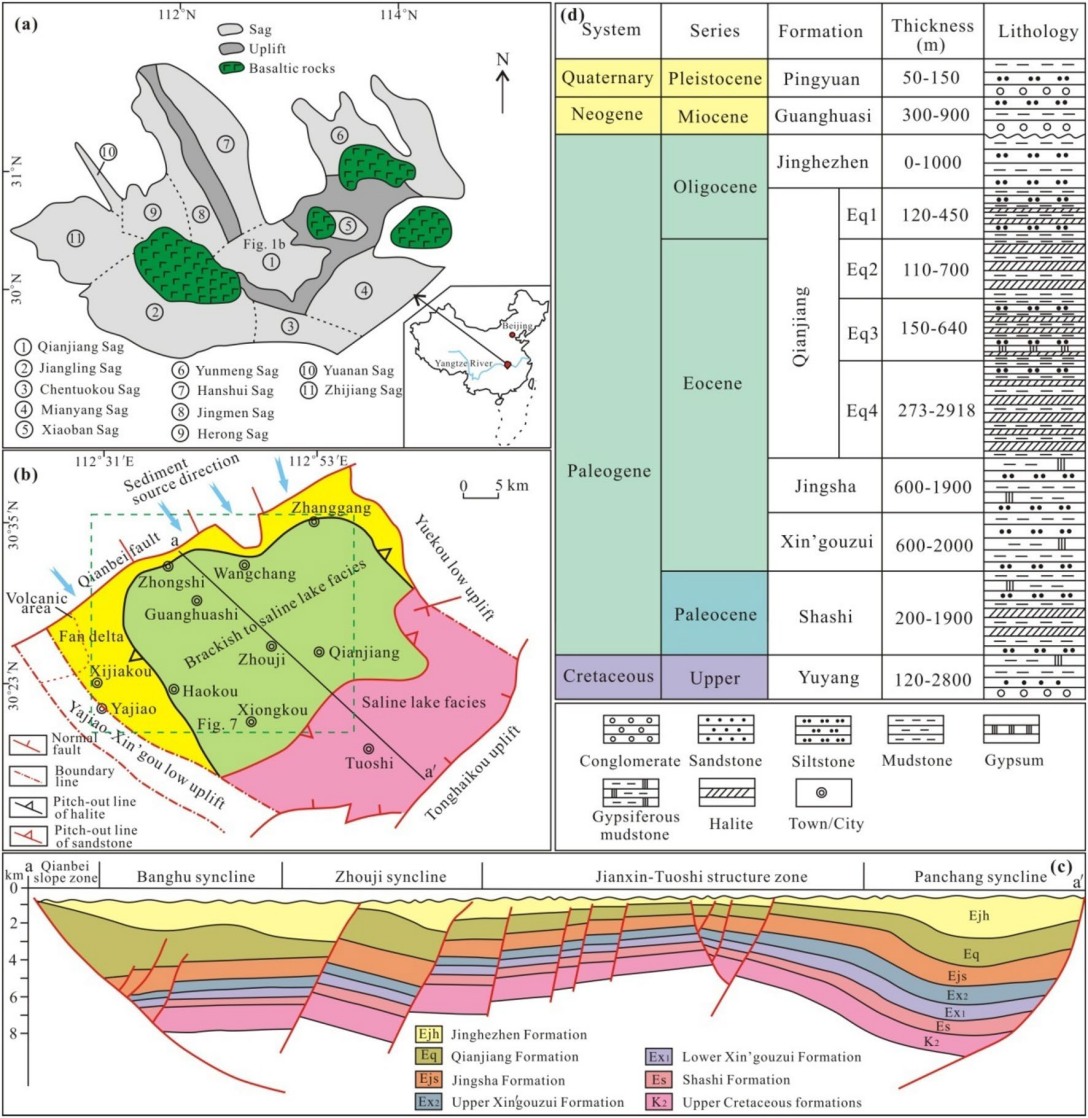
(**a**) Division of tectonic units of the Jianghan Basin^24^. (**b**) Sedimentary facies of the Qianjiang Formation of the Qianjiang Depression^24^. (**c**) Interpreted seismic profiles of the Qianjiang Depression. The location of the seismic profile is shown in (**b**). (**d**) Stratigraphic column of the Qianjiang Depression^24^.

The Jianghan Basin is a Cretaceous–Oligocene rift basin in central China, associated with subduction and rollback of the palaeo-Pacific Plate^21,22^. The basin underwent two salt-forming stages: Late Cretaceous to Early Eocene and Middle Eocene to Oligocene^23^. The Middle Eocene to Late Oligocene Qianjiang Depression is the subsidence and deposition center of the basin (Fig. 1a). The depression, covering a total area of approximately 2500 km^2^, is one of the most oil and gas rich regions in the Jianghan Basin^25^. It is bounded by the Qianbei fault to the northwest, the Yajiao–Xin’gou low uplift to the southwest, the Yuekou low uplift to the northeast, and the Tonghaikou uplift to the southeast (Fig. 1b). During the Middle Eocene to Early Oligocene, the intense faulting along the Qianbei fault led to the formation of a half-graben structural pattern that was deep in the northwest and shallow in the southeast. Thus, fan delta facies, transitional brackish to saline lake facies, and saline lake facies from northwest to southeast have been identified^26^.

The Qianjiang Depression is superimposed on the basement of pre-Sinian metamorphic rocks, Sinian to Middle Triassic marine sedimentary rocks and Upper Triassic continental sedimentary rocks. Paleogene volcanic rocks are distributed in the northwest of the basin^27^. The overlying basin strata, in ascending order, comprise the Upper Cretaceous Yuyang Formation, the Paleogene Shashi, Xin’gouzui, Jingsha, Qianjiang and Jinghezhen formations, and the Neogene Guanghuasi Formation (Fig. 1c). The Qianjiang Formation is a salt-bearing strata that yield abundant oil and gas resources, with a thickness of 4700 m in the depocenter. It is characterized by notable salt rhythmites and is composed of alternating halite, gypsum (or glauberite) and mudstone with siltstone and sandstone (Fig. 1d). The formation can be divided into four units from top to bottom: Eq1, Eq2, Eq3, and Eq4. Plenty of oilfield brines are produced in the sandstone reservoirs of the four stratigraphic intervals. The reservoirs are between 600 and 3900 m deep, with an average of 2200 m^18^.

## Sampling and methods

The oilfield water samples were collected in June and July 2021 from 155 oil wells in the Yajiao, Xijiakou, Guanghuasi, Zhouji, Wangchang, Zhongshi, Zhanggang, Xiongkou regions of the northern Qianjiang Depression. The ratio of water in the oil–water mixture is between 35% and 98%. The quantitative filter papers were used to separate the water from the oil at the sampling locations. Then, these water samples were filtered through 0.45 μm PTFE membrane filters and stored in the acid-washed HDPE bottles. Cation aliquots were acidified to pH < 2 with thermally distilled nitric acid and used for trace element analyses. Unacidified aliquots were used for anion analyses.

The main cations (K^+^, Na^+^, Ca^2+^, Mg^2+^, Li^+^, B^3+^, and Sr^2+^) of water samples were analyzed by inductively coupled plasma optical emission spectrometer (ICP-OES). Cl^−^, SO_4_^2−^ and Br^−^ were measured by ion chromatography (IC). HCO_3_^−^ and CO_3_^2−^ were determined by 0.05 M HCl titration (AT). The analytical errors for ICP-OES, IC and AT were 2‰, 5‰ and 3‰, respectively. The contour diagrams of TDS, Br, Li, and B were performed using Surfer 12.0 software.

## Results

**Figure 2:**
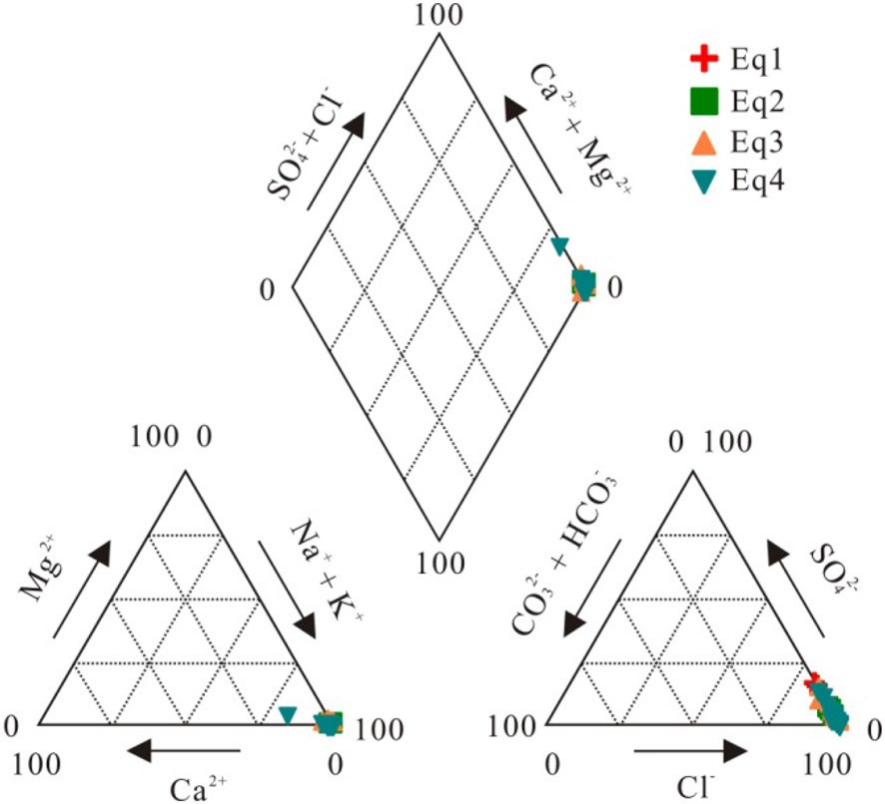
Piper diagram for oilfield brines in the Qianjiang Depression. The ratios plotted in the diagram are normality ratios.

The water chemistry data are given in the supplementary Table S1. The charge balance errors are less than 5.4% for all water samples. The brines are alkaline, with only a few acidic. They have total dissolved solids (TDS) ranging from 127 to 355 g/L. Based on the distribution pattern on a piper diagram, these brines can be classified as the Na–Cl or Na–Ca–Cl type (Fig. 2).

Na concentrations range from 46.10 to 132.90 g/L with a mean of 99.60 g/L. The K/Na molar ratios are less than 0.03. Most brine samples are characterized by Ca^2+^ > Mg^2+^. Cl contents range from 75.26 to 206.02 g/L with an average of 153.62 g/L. Concentrations of SO_4_^2−^ and HCO_3_^−^ are much lower than Cl^−^. The Na/Cl molar ratios are between 0.83 and 1.21, and the Cl/Br molar ratios are mostly between 478 and 2483 (Table S1). The Li concentration of the brines ranges between 7.56 and 150 mg/L, and the Mg/Li ratio is less than 11.65. These brines have high Br contents varying from 77.88 to 913 mg/L, with a mean of 329.51 mg/L. Boron concentrations range between 28 and 456.4 mg/L. The strontium contents show a large variation varying between 1.54 and 263.18 mg/L.

The brines from the Eq3 and Eq4 units have Li contents ranging from 9.98 to 150.00 mg/L, and are higher than the Eq1 and Eq2 waters which vary from 7.56 to 61.26 mg/L. B, Br, and Sr contents in each unit are relatively variable. Generally, the Eq3 and Eq4 waters have relatively high concentrations of Li, B, Sr, and Br.

## Discussion

### Chemical evolution process

**Figure 3:**
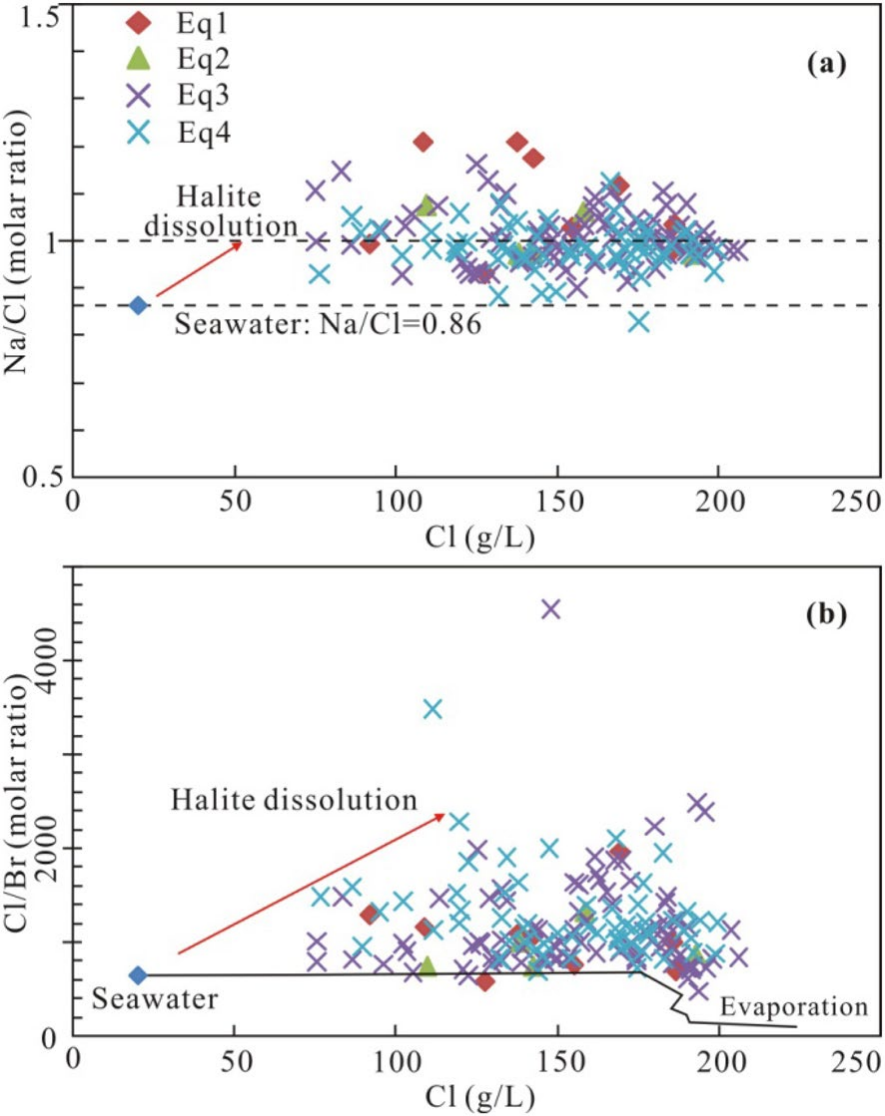
(**a**) Plot of Na/Cl molar ratios vs. Cl concentrations. (**b**) Plot of Cl/Br molar ratios vs. Cl concentrations in comparison to the seawater evaporation trajectory^31^.

**Figure 4:**
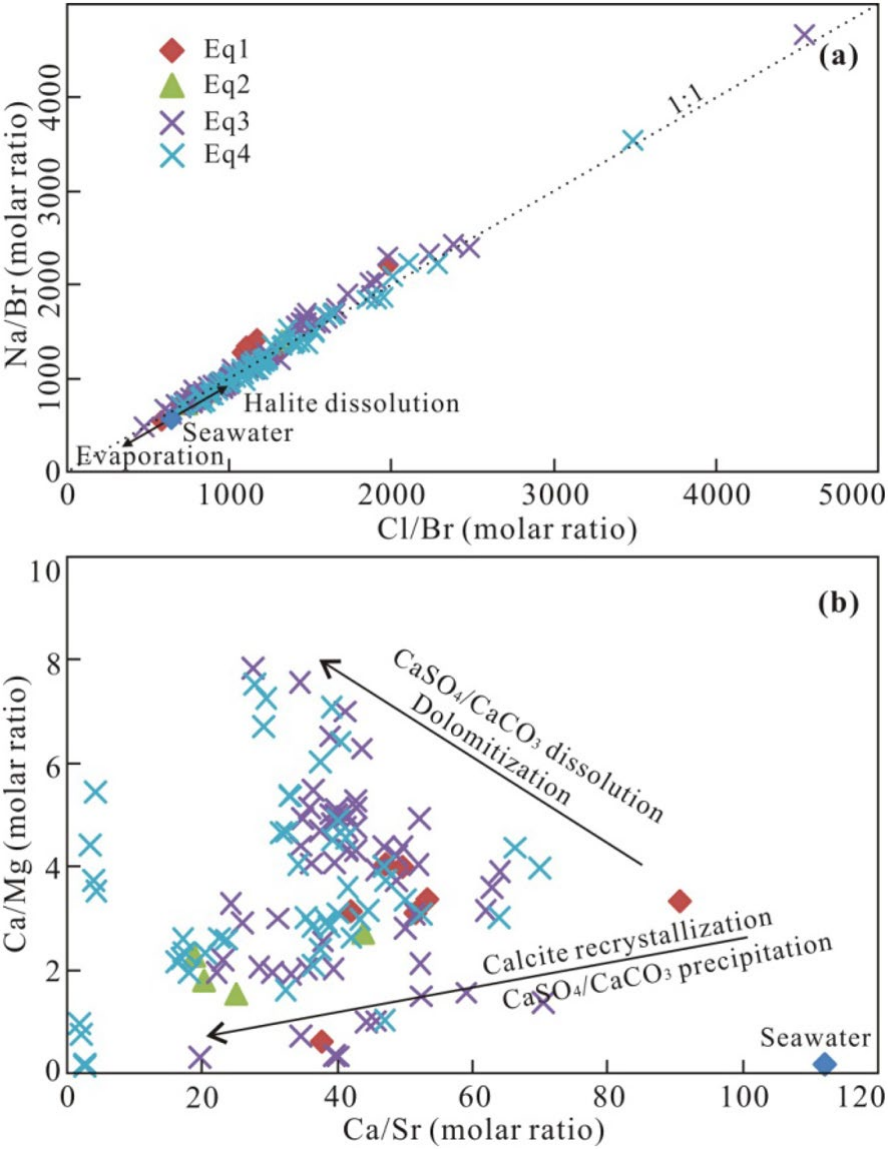
(**a**) Plot of Cl/Br vs. Na/Br molar ratios with trends for evaporating seawater and halite dissolution^32^. (**b**) Plot of Ca/Sr vs. Ca/Mg molar ratios with trends for calcite recrystallization and dolomitization processes^33^.

The salinity of formation waters in the sedimentary basins can be attributed to evaporation and/or evaporite dissolution^28,29^. Brines affected by halite dissolution have a Na/Cl molar ratio close to 1^30^, which is higher than that of seawater (0.86). The brine samples from the Qianjiang Depression have Na/Cl molar ratios ranging from 0.83 to 1.21 (Fig. 3a), most of which are close to 1, indicating dominant contributions from halite dissolution, as evidenced by the high Cl/Br ratios^31^ (Figs. 3b, 4a).

**Figure 5:**
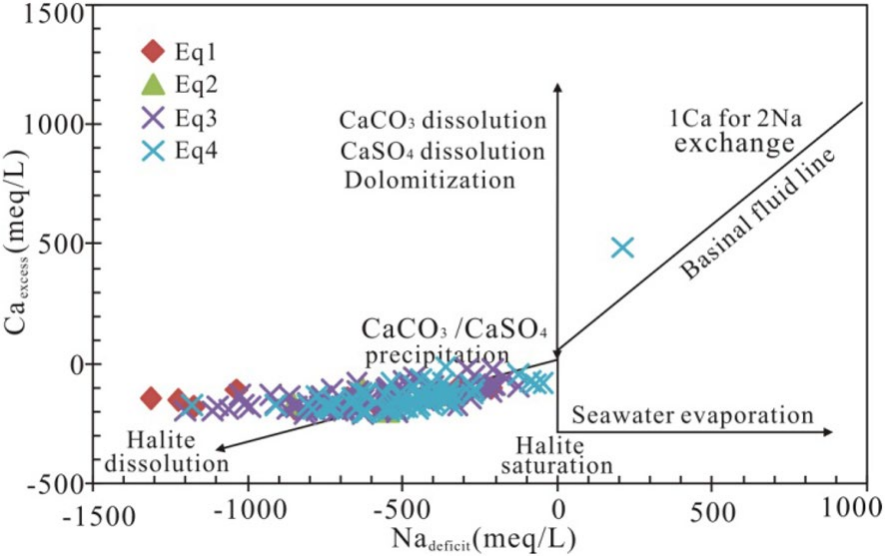
Plot of Na_deficit_ vs. Ca_excess_ for Eocene to Lower Oligocene oilfield brines^34^. The basinal fluid line (BFL) means the theoretical trend for the albitization of primary seawater.

The trends between Ca/Mg and Ca/Sr molar ratios (Fig. 4b) suggest dolomitization and calcite recrystallization processes^29,33^, which are also supported by the mineralogical analysis of reservoir rocks in the study area^20^. The linear relationship between the excess Ca and the Na deficit relative to seawater reference ratios can be used to trace multiple water–rock interactions in sedimentary basins^34^. Plotting of Ca excess and Na deficit of the Qianjiang brine samples shows multiple diagenetic processes (Fig. 5). Data for almost all samples are located near the trendline of halite dissolution, suggesting the origin affected by dissolution of halite and calcite or anhydrite cementation. Only one sample is plotted between the trendline of dissolution of CaSO_4_ and CaCO_3_ and dolomitization and the basinal fluid line (albitization), indicating the influence of multiple water–rock interactions.

### Spatial distribution pattern and origin of Li in brines

**Figure 6:**
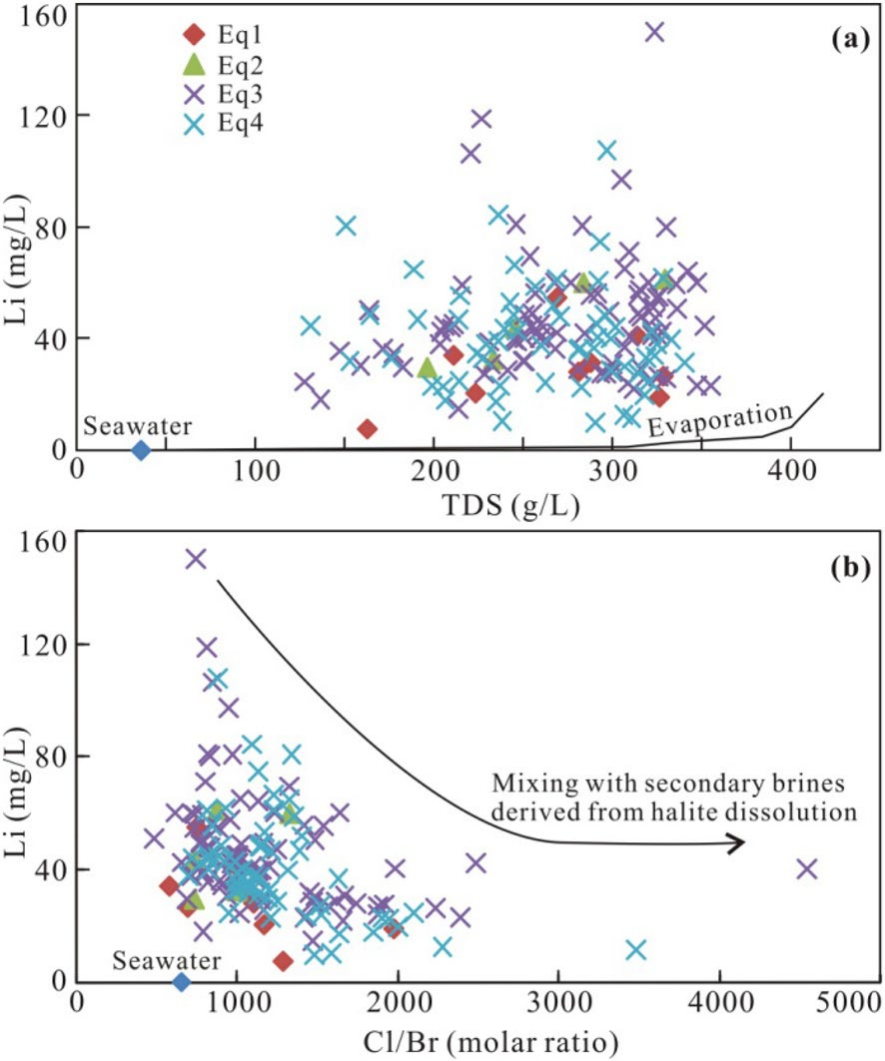
(**a**) Plot of TDS vs. Li concentrations in comparison to the seawater evaporation trajectory^31^. (**b**) Plot of Cl/Br molar ratios vs. Li concentrations.

Variations in the grades of Li in the Qianjiang brines can be revealed by plotting Li contents against salinity and Cl/Br molar ratios. Figure 6a shows a weak correlation, which is incompatible with the fact that the residual brines have higher Li concentrations^17^. A negative correlation is shown in the Fig. 6b, indicating that Li concentrations of these brines are likely diluted by the mixing of the fluids derived from halite dissolution.

**Figure 7:**
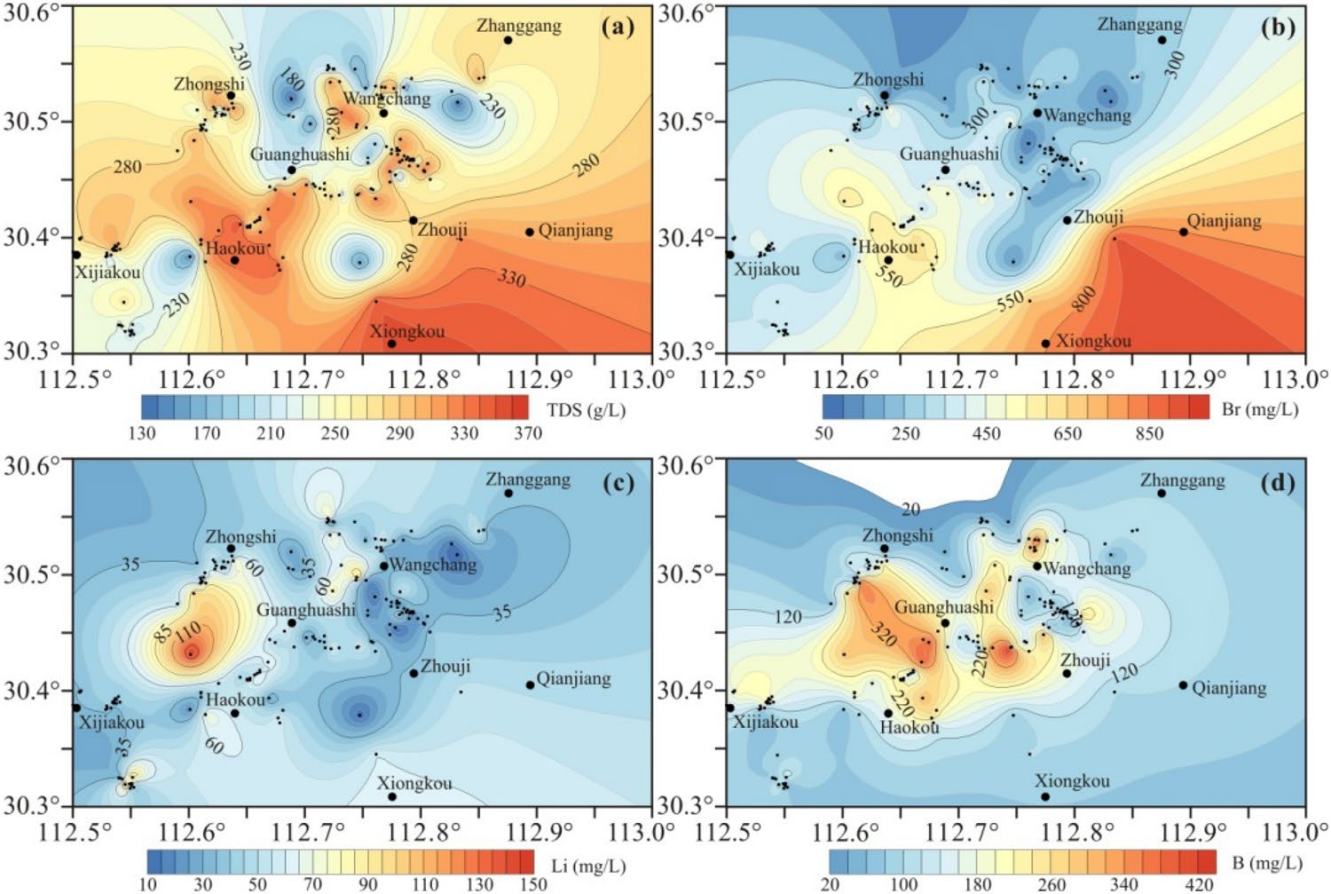
Contour diagrams of Li, B and Br concentrations and salinity of oilfield brines in the Qianjiang Depression. The area is shown in Fig. 1b.

Based on the contour diagrams of Li, B and Br contents and TDS of these brines in the Qianjiang Depression, some spatial distribution patterns seem to be revealed. The high-salinity brines are mainly distributed in the Haokou, Zhongshi, Wangchang and Xiongkou areas (Fig. 7a). The high-bromine brines are located in the Haokou, Xiongkou and Qianjiang areas (Fig. 7b). The high-lithium brines are distributed between the Haokou and Zhongshi regions (Fig. 7c), whereas the high-boron brines are located between the Haokou and Zhongshi regions, followed by areas near Zhouji and Wangchang (Fig. 7d). So, the distribution patterns for TDS and Br contents show similar variation feature, while those for Li and B contents exhibit different characteristics and have the affinity to the volcanic rocks to the northwest of the study area. It is possible that Br enrichment is related to evapo-concentration while special material sources are required for the mineralization of Li and B.

Weathering of igneous rocks and hydrothermal supplies associated with volcanic activity have been suggested to interpret the sources of Li-enriched brines^17,35–38^. The geographic distribution pattern for Li concentrations of these brines in the Qianjiang Depression, in combination with the widely developed volcanic rocks in the Jianghan Basin, indicates that Li enrichment in brines is more likely to be associated with geothermal sources during volcanic activity^39,40^.

## Conclusions

This study reports the results of the hydrochemical analysis of oilfield brines from the Qianjiang Formation of the Jianghan Basin, central China. The brines are of the Na–Cl or Na–Ca–Cl type and have Li contents ranging from 7.56 to 150 mg/L, with Mg/Li ratios less than 11.65.

High Na/Cl and Cl/Br molar ratios of these brines suggest contributions from halite dissolution. Plotting of Ca excess vs. Na deficit and Ca/Mg vs. Ca/Sr molar ratios indicates multiple diagenetic processes, including dolomitization, calcite or anhydrite cementation, halite dissolution and albitization. Variations in the grades of Li in brines have a weak correlation with the salinity. The Li contents of these brines may be diluted by the mixing of secondary fluids derived from halite dissolution. The spatial distribution for the concentrations of Li and B is different from those of TDS and Br and shows a geographic pattern, which is likely associated with geothermal supplies associated with volcanic activity.

## Supplementary Information

Supplementary Table S1.

## Data Availability

All data generated or analysed during this study are included in this published article and the supplementary information file (Table S1).
